# Altered epigenetic features in circulating nucleosomes in idiopathic pulmonary fibrosis

**DOI:** 10.1186/s13148-017-0383-x

**Published:** 2017-08-15

**Authors:** J. Guiot, I. Struman, V. Chavez, M. Henket, M. Herzog, K. Scoubeau, N. Hardat, B. Bondue, JL. Corhay, C. Moermans, R. Louis

**Affiliations:** 1 Pneumology Department, CHU Liège, Domaine universitaire du Sart-Tilman B35, 4000 Liège, Belgium; 2 Molecular Angiogenesis Laboratory, GIGA R, University of Liège, B34, 1 avenue de l hospital Sart-Tilman, Liège, Belgium; 3 Department of Clinical Hematology, CHU Sart Tilman, Liège, Belgium; 4 Belgian Volition SPRL, Rue du Seminaire 20A, 5000 Namur, Belgium; 5 Pneumology Department, Erasme hospital, université libre de bruxelles, Belgium Route de Lennik, 808, 1070 Brussels, Belgium

**Keywords:** Idiopathic pulmonary fibrosis, Epigenetic, Biomarkers, Nucleosome modifications, Interstitial lung disease

## Abstract

**Background:**

Idiopathic pulmonary fibrosis (IPF) is a progressive, fatal lung disorder of unknown origin with a highly variable and unpredictable clinical course. Polymorphisms and environmentally induced epigenetic variations seem to determine individual susceptibility to the development of lung fibrosis.

**Methods:**

We have studied circulating epitopes on cell-free nucleosomes (cfnucleosomes) in 50 IPF patients. We have compared untreated IPF (*n* = 23) with IPF receiving antifibrotic therapy (*n* = 27) and healthy subjects (HS) (*n* = 27). We analyzed serum levels of five cfnucleosomes including bound HMGB1 (nucleosomes adducted to high-mobility growth protein B1), mH2A1.1 (nucleosomes containing the histone variant mH2A1.1), 5mC (nucleosomes associated with methylated DNA), and H3K9Ac and H3K27Ac (nucleosomes associated with histone H3 acetylated at lysine 9 or 27 residue).

**Results:**

Our findings showed that serum levels of bound HMGB1, mH2A1.1, 5mC, H3K9Ac, and H3K27Ac were significantly lower in IPF patients than in HS (*p* < 0.001, *p* < 0.001, *p* < 0.01, *p* < 0.001, and *p* < 0.0001, respectively). Moreover, we found differences in epigenetic profiles between untreated IPF patients and those receiving anti-fibrotic therapy with mH2A1.1 and 5mC being significantly lower in untreated than in treated patients (*p* < 0.01 and *p* < 0.05, respectively). Combination of four cfnucleosomes (HMGB1, 5mC, H3K9Ac, and H3K27Ac) allow to discriminate IPF vs HS with a good coefficient of determination (*R* ^2^ = 0.681). The AUC for the ROC curve computed by this logistic regression was 0.93 (*p* < 0.001) with 91% sensitivity at 80% specificity.

**Conclusion:**

Our observations showed that cfnucleosomes (bound HMGB1, mH2A1.1, 5mC, H3K9Ac, and H3K27Ac) might have potential as biomarkers for diagnosis and treatment response. These results deserve further validation in longitudinal cohorts.

## Background

Idiopathic pulmonary fibrosis (IPF) is a progressive, fatal lung disorder of unknown origin with a highly variable and unpredictable clinical course [1–3]. Post-transcriptional modifications and gene-environment interactions (e.g., smoking, gastro-oesophageal reflux, and viral infections) appear to be key factors in IPF pathophysiology [4–7]. Polymorphisms and environmentally induced epigenetic variations seem to be implicated in individual susceptibility to fibrosis development and disease progression [8]. Profound phenotype changes occur in the lung; hence, alveolar epithelial cells become hyperplasic and potentially mesenchymal cells while fibroblasts present myofibroblast characteristics. Differential expression patterns of mRNA and microRNA (correlated with epigenetic modifications) have been observed in IPF compared to healthy subjects (HS) [9]. This suggests that epigenetic marks may be mediators of environmental exposure effects in genetically predisposed individuals and may lead to the transcriptional changes that occur in IPF [8]. Although little is known about epigenetic alterations in IPF [4, 10], experimental evidence has shifted the pathophysiological paradigm from chronic inflammation towards abnormal epithelial wound repair with repeated epigenetic mutational challenge [4].

Nucleosomes are the basic unit of chromatin consisting in a 147-bp DNA strand wrapped around a core of eight histone protein cores. During cellular damage such as apoptosis or necrosis, chromatin is fragmented into oligo- or mono-nucleosomes which are released into the blood stream [11, 12]. During the last decade, cfnucleosomes have appeared as novel non-invasive biomarkers that could inform on disease potential progression. However, the level of cfnucleosomes itself may not have optimal diagnostic value, due to their non-specific release as it may occur in cancer and acute and chronic non-malignant inflammatory conditions [13]. Nonetheless, specific epigenetic changes could be more informative. Histone and DNA are both subjected to epigenetic modifications. Epigenetic nucleosome modifications including histone acetylation, histone methylation, or DNA methylation play fundamental roles in gene regulation and expression [14]. Histone acetylation reduces the affinity between histones and DNA, thereby facilitating gene transcription. Histone methylation or DNA methylation has been mostly associated with a repression of gene transcription even if activation has also been described. In addition, nucleosome can be associated with adduct proteins such as high-mobility group protein 1, a protein involved in inflammation and in pulmonary fibrosis [15]. As a new approach for diagnosis and monitoring of IPF, we have studied the relevance of some nucleosome epigenetic features including histone and DNA modifications as well as histone variants or nucleosome adducts in serum samples of treated and untreated IPF patients.

## Methods

### Subject characteristics

Patients were recruited from the ambulatory care polyclinics of both Liège University Hospital and Erasme University Hospital (Brussels). Patients were divided into two groups: patients with untreated IPF (*n* = 23) and patients with treated IPF (*n* = 27: 18 treated with pirfenidone: 9.9±7.4 months; 9 treated with nintedanib: 14.3±15.8 months (mean ± SD)). The diagnosis of (definite) IPF was made according to the international recommendations of the ATS [1] using the respiratory function test, HRCT scan (probable UIP pattern), bronchoalveolar lavage (when available), as well as the clinical history of the patient. All other causes of interstitial lung disease (such pneumoconiosis, hypersensitivity pneumonitis, pneumonia associated with connective tissue disease, or drug-induced interstitial lung disease) were excluded. Diagnosis was based on the combination of the above results. All cases were discussed by an interstitial lung disease multidisciplinary group of pulmonologists, rheumatologists, radiologists, pathologists, and occupational medicine and pulmonary rehabilitation specialists. Sixteen patients underwent a surgical biopsy and seven patients trans-bronchial cryobiopsies. Twenty-seven patients were treated with a specific treatment of IPF (pirfenidone (*n* = 18) or nintedanib (*n* = 9)). HS were recruited from Liège university hospital by advertising in the waiting room. None showed any symptoms of a respiratory disease, and all had normal spirometric values with predicted forced expired volume in one second (FEV1) > 80% and FEV1/FVC ratio > 70%. Patients treated with histone deacetylase activators like theophylline were also excluded.

The protocol was approved by the ethics committee of CHU of Liège, and all subjects gave written consent before their enrolment (Belgian number: B707201422832; ref.: 2014/302).

### Cell-free circulating nucleosome measurements

Venous blood was collected at enrolment in Vacutainer tubes (for serum sampling) from an antecubital site. Ten nucleosome epitopes, corresponding to nucleosomes associated methylated DNA (5-methylcytosine (5mC)); nucleosomes associated with histone modifications H3K9Ac, H3K27Ac, H4PanAc, H3K9me3, H4K20me3, H3K4Me2, or pH2AX; nucleosomes containing the histone variant mH2A1.1; and nucleosomes adducted to high-mobility growth protein B1 (HMGB1), were analyzed in the serum of IPF patients and controls.

Epitopes on cfnucleosome levels were measured using Nu.Q™ enzyme-linked immunosorbent assays (ELISA) (Belgian Volition SPRL, Namur, Belgium) performed according to the manufacturer’s instructions [16, 17]. In brief, serum samples (10 μl in duplicate) were incubated for 2 h 30 min at room temperature in a 96-well microtiter plate coated with a monoclonal anti-nucleosome antibody. After washing steps, the level of cfnucleosomes containing particular epigenetic feature were quantified by adding into the well a biotinylated detection antibodies directed to specific epigenetic feature of the nucleosomes under investigation (incubation 90 min at room temperature). The wells were washed and streptavidin-horseradish peroxidase (HRP) was added. After incubation for 30 min at room temperature, the wells were washed and a peroxidase substrate-2,2′-azino-bis-(3-ethylbenzothiazolonine-6 sulfonic acid) was added. The optical densities of the wells were read with an X-Mark Microplate spectrophotometer (BioRad).

## Statistical analysis

All the statistical analyses were performed with the SPSS Statistics 24 software (IBM). For the different clinical groups, the mean value of duplicate measurements was used. Samples with a coefficient of variation (%CV) above 20% between its duplicates were repeated. The mean values were taken into consideration for the statistical analysis only if it reached the criteria: CV < 20. The distribution of the marker levels is given as median, interquartile, and total ranges. Comparisons of cfnucleosomes between the groups were performed by Kruskall-Wallis followed by Mann-Whitney *U* test in case of statistical significances. Area under the receiver operating characteristic (ROC) curves and sensitivities at defined specificities were calculated to test the performance of the biomarkers for differential diagnosis. Binary multiple logistic regressions were used to validate the statistical significance of the cfnucleosome biomarkers and to evaluate their capacity to predict disease. *p* values < 0.05 were considered as statistically significant.

## Results

### Subject demographic and functional characteristics

The demographic functional and treatment characteristics are given in Table 1. The average age of IPF patients was 72 ± 11 years for untreated patients vs 68 ± 9 years for treated patients. Spirometric values were moderately lower and comparable in both the treated and untreated IPF while diffusion lung capacity for CO (DLCO) was sharply reduced in both IPF groups.

**Table 1:** Subject characteristics

	Healthy subjects *n* = 27	Untreated IPF *n* = 23	Treated IPF *n* = 27
Age, years	60(9)	72(11)**	68(9)*
Gender (M/F)	14/13	20/3	19/8
Height, cm	170(9)	171(10)	170(9)
Weight, kg	74(12)	76(15)	78(10)
BMI, kg/m^2^	26(3)	26(4)	27(3)
Smokers (NS/FS/S)	6/17/4	6/13/4	7/20/0
FEV1 post-BD, %pred.	104(14)	75(14)***	67(13)***
FVC post-BD, %pred.	112(20)	74(14)***	66(16)***
FEV1/FVC post-BD, %	78(5)	78(11)	80(6)
TLC, %pred.	nd	70(13)	68(15)
RV, %pred.	nd	71(32)	82(27)
DLCO, %pred.	nd	39(13)	38(13)
KCO, %pred.	nd	62(13)	70(20)
ICS (yes/no)	0/27	1/22	0/27
OCS (yes/no)	0/27	0/23	0/27
Treatment with pirfenidone/nintedanib	nd	nd	18/9

Data are expressed as mean (SD)

*nd* not determined, *NS* non-smoker, *FS* former smoker, *S* smoker, *FEV1* forced expired volume in one second, *FVC* forced vital capacity, *TLC* total lung capacity, *DLCO* diffusion lung capacity for CO, *KCO* DLCO/alveolar ventilation, *ICS* inhaled corticosteroids, *OCS* oral corticosteroids

*p < 0.05, **p < 0.001, ***p < 0.0001 compared to healthy subjects

### Circulating cell-free nucleosomes

Epigenetic profiles of cfnucleosomes in subjects with untreated IPF, treated IPF, or HS were investigated using ELISA-based Nu.Q™ assays. Ten epigenetic features of serum cfnucleosomes were analyzed. The receiver operator characteristic (ROC) curves for each nucleosome assay in the untreated IPF group vs HS were established (data not shown). The area under the curve (AUC) for the individual ROC curves varied from 0.54 to 0.85 (Table 2).

**Table 2:** Single cfnucleosome AUC and sensitivity at 80% specificity

Nu.Q™ assay	IPF (untreated) vs healthy	
	AUC	% sensitivity at 80% specificity
mH2A1.1	0.85	86
5mC	0.83	78
HMGB1	0.81	74
H3K27Ac	0.79	74
H3K9Ac	0.77	61
H4K20Me3	0.70	50
pH2AX	0.66	39
H4Pan(Ac)	0.57	13
H3K4Me2	0.57	14
H3K9Me3	0.54	13

cfnucleosome features between healthy subject (*n* = 27) and untreated (*n* = 23) idiopathic pulmonary fibrosis patients. The receiver operator characteristic (ROC) curve analysis is calculated at 80% specificity. All serum nucleosome modifications were analyzed by ELISA Nu.Q™ assays

*AUC* area under the curve

**Fig. 1:**
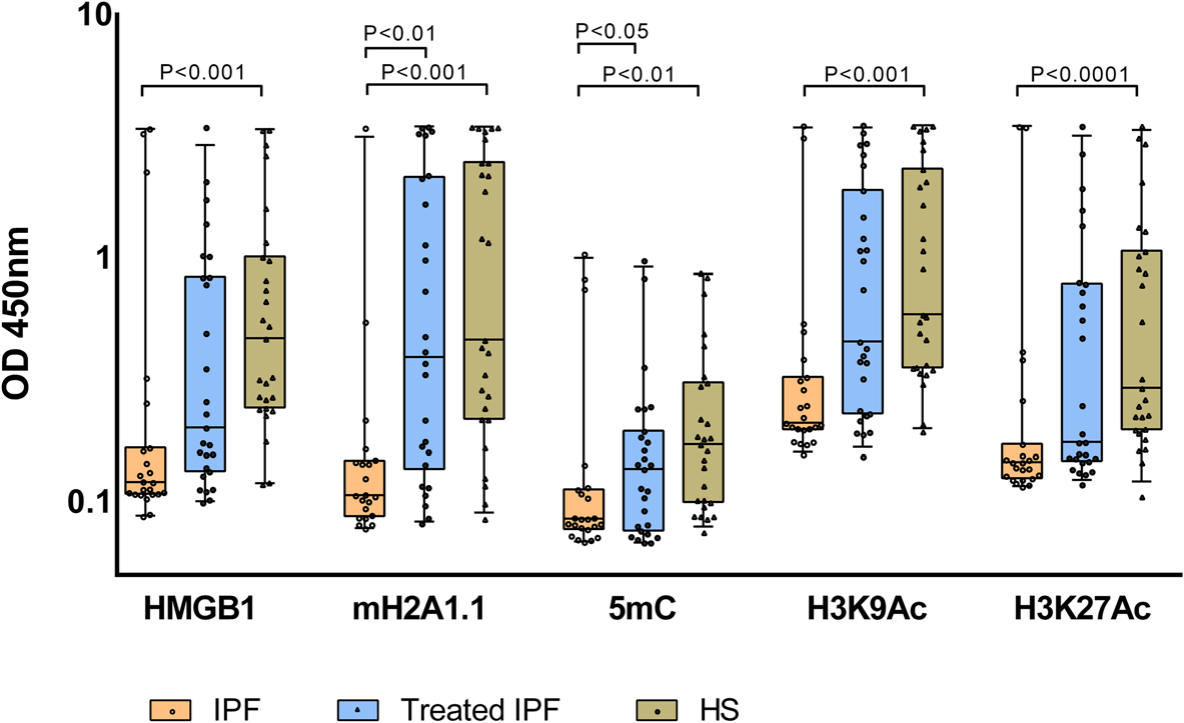
Serum cfnucleosome modifications in IPF. Discrimination of five Nu.Q™ assays for untreated IPF (*n* = 23), treated IPF (*n* = 27), and healthy subjects (HS) (*n* = 27). Treatment is pirfenidone (*n* = 18) and nintedanib (*n* = 9). Nu.Q™ assays were performed from serum samples. Significant separation between the untreated IPF and healthy controls was achieved with pre-processed ELISA data from the five cfnucleosome biomarkers. Significant discrimination between the treated and untreated patients was achieved from the two cfnucleosome biomarkers. *p* values were determined by the Mann-Whitney *U* test. *Box plots* indicate the median and 25th and 75th percentiles. *Whiskers* indicate the 10 and 90% percentiles and dot values falling outside the 10th and 90th percentiles. *IPF* idiopathic pulmonary fibrosis, *OD* optical density

Of note, diagnostic sensitivity at 80% specificity for the five best nucleosome biomarkers ranged from 61 to 86% (Table 2). Remarkably, the level of these five cfnucleosome features, namely, HMGB1, mH2A1.1, 5mC, H3K9Ac, and H3K27Ac were significantly lower in untreated IPF than in HS (Fig. 1). Box plots derived from the best five individual assays based on the AUC are shown in Fig. 1.

Interestingly, 5mC and mH2A1 levels allowed to discriminate between untreated and treated IPF patients (*p* < 0.05 and *p* < 0.01, respectively) (Fig. 1). Remarkably, in our study, specific anti-fibrotic therapies significantly modified these two markers in treated IPF patients bringing them close to HS values (Fig. 1).

Correlation studies between lung function indices and cfnucleosomes showed a positive correlation between DLCO/VA (%pred) and H3K27Ac or H3K9Ac (*r* = 0.313, *p* = 0.049; *r* = 0.341, *p* = 0.032, respectively).

**Fig. 2:**
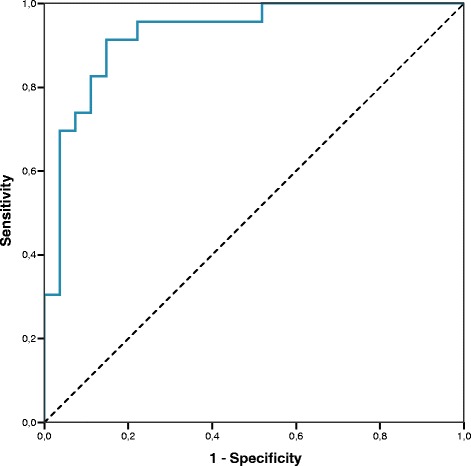
A model with four cfnucleosome biomarkers discriminates IPF vs healthy patients. ROC curve for the discrimination of untreated IPF vs healthy subjects. The AUC for a model of four cfnucleosome biomarkers (HMGB1, 5mC, H3K9Ac, H3K27Ac) reached 0.928 (*p* < 0.001) with a sensitivity of 91% at 80% specificity

In addition, we assessed if an increase in the diagnostic value could be observed by combining levels of cfnucleosome modifications. Statistical significance was calculated for several combinations using binary logistic regressions and the best combination was selected. This study revealed a highly statistically significant (*p* < 0.001) combination of four cfnucleosomes: HMGB1, 5mC, H3K9Ac, and H3K27Ac with good coefficient of determination (*R* ^2^ = 0.681). In this combination, each individual predictor was already highly statistically significant. The AUC for the ROC curve computed by this logistic regression was 0.93 (*p* < 0.001) with 91% sensitivity at 80% specificity for the discrimination of IPF patients vs HS (Fig. 2). We also notice that this analysis was gender independent (all *p* values > 0.1). Pearson correlation analysis between age and levels of cfnucleosome modifications showed only a marginal correlation between the two parameters (*r* coefficient of 0.299, 0.330, 0.210, 0.265, and 0.281 for HMGB1, 5mC, H3K9Ac, H3K27Ac, and mH2A1.1, respectively).

## Discussion

We have investigated the clinical diagnostic accuracy for IPF using ELISA tests for epigenetic epitope variation in cfnucleosomes in small volumes of serum as a readily available and non-invasive sample type.

Our finding showed a reduced level of cfnucleosomes associated with HMGB1. HMGB1 is loosely bound to the chromatin during interphase and mitosis and has a rapid turnover between its chromatin-bound and soluble states. On the contrary, hyperacetylation occurring in apoptosis favors its chromatin-bound state, thereby preventing HMGB1 release [17]. Although HMGB1 implication in inflammation and tissue repair has been well described, its role in IPF seems to be complex and need further elucidation [18].

Our results showed lower circulating chromatin-bound HMGB1 in IPF than in normal subjects. This suggests increase HMGB1 tendency towards its soluble form in IPF as it has been previously described [15]. Therefore, extracellular HMGB1 in IPF could play a protective role in preventing abundant release of nucleosomes as it was previously described during apoptosis and repair process in lung remodeling [19].

Recent data have shown high histone deacytelase activity in fibroblasts and myofibroblasts from patients with IPF [20, 21]. Our results are in agreement with that finding since we found lower levels of histone H3 acetylated markers (H3K9Ac and H3K27Ac) in untreated IPF patients. Interestingly, in the current study, patients treated with anti-fibrotic drugs display cfnucleosome epitope profile comparable to HS, which suggests that these drugs may partially restore normal levels of histone H3 acetylation. Moreover, there is a positive correlation between hypoacetylation and the severity of the disease assessed by the DLCO/VA quotient, which is known to be a good marker of the alveolo-capillar membrane function. However, this correlation is not very strong and has to be confirmed in a larger prospective study.

Acetylation homeostasis in nucleosomes is the result of a fine equilibrium between histone acetyl transferases (HAT) and histone deacetylases (HDAC). Disruption of this balance either by an increase of HDAC or a decrease of HAT or both has been claimed to be involved in the pathophysiologic process of IPF [15]. Given that increased acetylation by HDAC inhibition reduces collagen production in different fibroblast types, new anti-fibrotic drug (suberoylanilide hydroxamic acid) acting as a histone deacetylase inhibitor (HDACi) appeared to be a promising therapeutic option in IPF by reducing collagen deposition [22, 23].

Methylation is known to be deregulated in IPF [9]. IPF-associated differentially methylated regions occurred primarily in promoter regions of genes involved in biological processes such as cellular assembly and organization, cellular growth and proliferation, gene expression, and cell death. All of these processes are reported to be implicated in IPF pathogenesis [9]. Our study also highlighted that the level of cfnucleosome associated methylated DNA (5-methylcytosine) is significantly reduced in untreated IPF compared to HS. Moreover, specific anti-fibrotic therapies restore 5mC to levels closed to those seen in HS. Therefore, nucleosome-associated 5mC could potentially be useful for therapeutic monitoring or prognostic factor to treatment response.

Likewise, we identified a significant reduction in the level of the cfnucleosome carrying the histone variant mH2A1.1 in untreated IPF while those patients receiving anti-fibrotic drugs had levels similar to HS.

Thinking beyond a single biomarker, we have assessed the value of combining several cfnucleosome modifications. After performing specific logistic regression comparing HS to untreated IPF, we found that a model of four Nu.Q™ cfnucleosome biomarkers (5mC, HMGB1, H3K27ac, and H3K9ac), reached an AUC of 0.93 in a ROC curve analysis (80% specificity and 91% sensitivity), which holds promise for use in clinical practice.

## Conclusions

In conclusion, we have identified new blood-based cfnucleosome markers in untreated IPF patients. Intriguingly, some of those biomarkers tend to normalize in the patients receiving anti-fibrotic treatment. We believe these cfnucleosomes may have potential as diagnostic and treatment response tools. This observation warrants further validation in larger longitudinal cohorts to confirm their value as biomarkers in IPF.

## Abbreviations

5mC: 5-Methylcytosine
ATS: American Thoracic Society
AUC: Area under the curve
CV: Coefficient of variation
DLCO: Diffusion lung capacity for CO
FEV1: Forced expired volume in one second
FVC: Forced vital capacity
HMGB1: High-mobility group protein 1a
HRCT: High-resolution computed tomography
HRP: Horseradish peroxidase
HS: Healthy subjects
IPF: Idiopathic pulmonary fibrosis
KCO: Diffusion lung capacity for CO/alveolar ventilation
ROC: Receiver operating characteristic
SD: Standard deviation
UIP: Usual interstitial pneumonia
